# Capillaric field effect transistors

**DOI:** 10.1038/s41378-022-00360-8

**Published:** 2022-03-21

**Authors:** Claude Meffan, Julian Menges, Fabian Dolamore, Daniel Mak, Conan Fee, Renwick C. J. Dobson, Volker Nock

**Affiliations:** 1 Department of Electrical and Computer Engineering, University of Canterbury, Christchurch, 8041 New Zealand; 2 Department of Microengineering, Kyoto University, 615-8540 Kyoto, Japan; 3 School of Biological Sciences, University of Canterbury, Christchurch, 8041 New Zealand; 4 Biomolecular Interaction Centre, School of Biological Sciences, University of Canterbury, Christchurch, 8041 New Zealand; 5 School of Product Design, University of Canterbury, Christchurch, 8041 New Zealand; 6 Department of Biochemistry and Molecular Biology, Bio21 Molecular Science and Biotechnology Institute, University of Melbourne, Parkville, VIC 3010 Australia; 7 The MacDiarmid Institute for Advanced Materials and Nanotechnology, Wellington, 6140 New Zealand

**Keywords:** Microfluidics, Nanofabrication and nanopatterning

## Abstract

Controlling fluid flow in capillaric circuits is a key requirement to increase their uptake for assay applications. Capillary action off-valves provide such functionality by pushing an occluding bubble into the channel using a difference in capillary pressure. Previously, we utilized the binary switching mode of this structure to develop a powerful set of fundamental fluidic valving operations. In this work, we study the transistor-like qualities of the off-valve and provide evidence that these structures are in fact functionally complementary to electronic junction field effect transistors. In view of this, we propose the new term capillaric field effect transistor to describe these types of valves. To support this conclusion, we present a theoretical description, experimental characterization, and practical application of analog flow resistance control. In addition, we demonstrate that the valves can also be reopened. We show modulation of the flow resistance from fully open to pinch-off, determine the flow rate–trigger channel volume relationship and demonstrate that the latter can be modeled using Shockley’s equation for electronic transistors. Finally, we provide a first example of how the valves can be opened and closed repeatedly.

## Introduction

Capillarics is a growing field within microfluidics that offers great potential for point-of-care testing due to its use of capillary action-driven, preprogrammed chips that can be fabricated in large numbers^1–3^. Without the need for auxiliary pumping and valving operations, complex operations can be scaled down easily, offering lab-on-a-chip level functionality in cheap, portable, and user-friendly devices^4,5^. These advantages have led to capillarics being used for a wealth of new applications in recent years, particularly in the biosensing space^6–18^. One fundamental requirement for the fully autonomous operation of capillaric circuits continues to be the availability of basic valving operations that do not need any external power source. Until recently, this was limited to two-level trigger valves^1,2,19^ or externally controlled valves^20,21^.

**Fig. 1:**
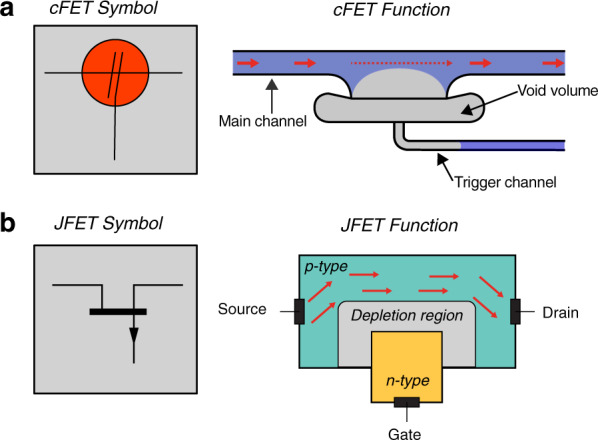
Construction and operational principle of the off-valve as a capillaric field effect transistor (cFET). **a** Circuit symbol, structure and operational principle of the cFET. An air bubble is expanded into the main channel via a trigger channel to control flow resistance in the former. **b** Symbol and basic operation of an electronic JFET. In this structure, the depletion region reduces the cross-section of the conducting channel, restricting electrical current flow. Despite different physical mediums, the similar geometries of these structures allow for significant theoretical and operational overlap

By expanding available valving options, we recently reported a capillary action off*-*valve structure that uses capillary pressure to inflate an occluding air bubble into a microfluidic channel, thus preventing further flow^22^. While trapped bubbles have been used for various applications in pressure-driven microfluidics^23^, including to alter flow dynamics and mixing^24^ and to realize hydraulic capacitors^25^, they have not yet been extensively used in capillarics. To fill this gap, we demonstrated autonomous capillary-action fluidic valving, with no need for external instrumentation, and a variety of fundamental valving operations, including use to provide feedback, metering, and logic operations^26^. As eluded to in our previous work, the off*-*valves hold the potential for more transistor-like switching and resistance tuning. This stems from the construction and operational principle of the off*-*valve, which are shown in Fig. 1 in comparison to an electronic junction field effect transistor (JFET)^27,28^. In the current work, we demonstrate that the off*-*valve can indeed be used to control the flow resistance of the main channel in nonbinary ways and that it can also oscillate. The particular operating principle of the off-valve is different from that of other fluidic transistors, such as the pressure-controlled field effect transistor (pFET)^29^, in that it acts on a continuous liquid volume and actually modulates the volume flow. We thus propose the introduction of the term capillaric field effect transistor (cFET) for the off*-*valve to reflect this new functionality, the role of the junction, and similarity to an electronic JFET and pFET.

As shown in Fig. 1a, the off*-*valve/cFET device consists of three structures: the main channel, a trigger channel, and a void volume. The main channel is the fluidic channel being switched, whereas the trigger channel is a smaller channel providing the capillary pressure used for switching. This pressure is delivered via the void volume, which acts as an isolation structure, insulating the main and trigger channels from each other. During off*-*valve operation, liquid wets through the main channel and across the large opening at the interface with the void volume. As indicated in Fig. 1a, the liquid does not flow into the void volume because of the height difference between the two structures and the sharp geometric edge created by this condition^30^. This leaves a large, low-pressure meniscus spanning the opening. When the trigger channel subsequently fills with liquid, air is displaced. The high-pressure trigger channel meniscus forces the low-pressure main channel meniscus into the main channel. This locates an occluding bubble into the main channel, restricting fluid flow through it. When the occluding bubble reaches the opposite wall of the main channel, fluid flow is pinched off.

For comparison, an electronic JFET and its operation principle are also shown in Fig. 1b. Both are three-terminal devices that feature a conductive channel, either liquid or electronic. In the case of the electronic JFET, a field effect applied from a control terminal forces a nonconductive volume into the main channel, reducing its cross-sectional area and increasing its resistance. The electric field depleting the semiconductor of p-type charge carriers in the JFET is comparable to the pressure field created by the trigger channel depleting the main channel of fluid in the cFET.

The conceptual symmetry an off*-*valve has with an electronic JFET means that the many existing theoretical transistor frameworks and circuits used in electronics may have practical implementations in microfluidics. To realize the microfluidic equivalents of transistor circuits, the controllability and behavior of the off-valves need to be better understood. The primary goal of this work is thus to better understand the transistor-like qualities that the off-valve possesses so that the mature circuit design concepts already existing in electronics can be applied to microfluidics. Two areas of interest for potential off*-*valve transistor functionality^27^ are analog resistance control and reversable operation. Previously, the valve was not demonstrated to control the flow resistance of the main channel in nonbinary ways. In addition, the structure could not oscillate—it could not be reopened and then reclosed. In some sense, this lack of functionality is intrinsic to capillary devices, as all operations are hardwired for single use^31^. There may be options to bypass this in future setups, but the number of uses will always be limited to the operations hardwired on the chip.

An important characteristic that the capillary off-valve and electronic transistors share is that they can exert a continuous controlling force on the flow resistance of the main channel using a third (fluidic) terminal. This “exerting control” property in electrical transistors is the transfer conductance or the transconductance of the device^27^. This concept defines the size of the conductance change of the main channel, which is transferred or exerted from the control terminal. When the transistor is integrated into a circuit, the transconductance can then be used to calculate circuit gain. The transconductance of a capillary off*-*valve can be experimentally determined and used with existing transistor models to predict behavior.

In the following, we develop a theoretical model for the closing dynamics of the off*-*valve, show the flow rate–volume relation, adapt the JFET Shockley equation^27,28^ for this capillary structure, demonstrate that a capillary off*-*valve can be reopened, and discuss the relevance and future applications for capillary transistors. We use the term capillaric FET, or cFET, from this point to reflect this new evidence.

The theoretical basis for many capillary action structures is the Young–Laplace equation (Eq. 1). This describes a general capillary pressure, *P*_cap_, created by a liquid–gas interface^32^. In this work, the Young–Laplace equation for square microchannels and for principal meniscus curvature is used:

1 $$\begin{array}{ll}p_{\rm{cap}} &= - \gamma \left[ {\frac{{\cos \theta _{\rm{roof}} + .\cos \theta _{\rm{floor}}}}{h} + \frac{{\cos \theta _{\rm{left}} + \cos \theta _{\rm{right}}}}{w}} \right]\\& = - \lambda \left( {\frac{1}{{R_{\rm{horizonatal}}}} + \frac{1}{{R_{\rm{vertical}}}}} \right)\end{array}$$

where $\gamma$ is the liquid surface tension, *θ* is the water contact angle with the channel roof, floor, and left and right walls, respectively, $h$ is the height of the microchannel, $w$ is the width of the microchannel, and $R_{\rm{horizontal}}$ and $R_{\rm{vertical}}$ are the principal radii of curvature of the meniscus. This equation is used in the following section to derive a model for the transient dynamics of the cFET.

## Results

### Transient dynamics of the cFET

**Fig. 2:**
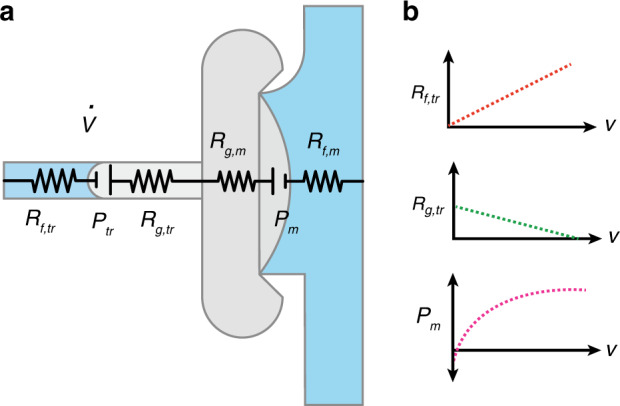
Electrical analogy model, and fluidic and gas resistances of the cFET. **a** Dynamic electrical analogy model for the closing behavior of a cFET. The model is overlaid on a representation of the physical structure to show the physical quantities they represent. Two capillary pressures, *P*_tr_ and *P*_m_, and four resistances, *R*_f,tr_, *R*_g,tr_, *R*_g,m_, and *R*_f,m_, are used to model the cFET behavior. **b** The fluid and gas resistances, and the main channel capillary pressure change as a function of the displaced trigger channel volume. Note: These graphs show the expected form of these properties only

It is important to develop a thorough theoretical understanding of the dynamics of the cFET to develop a model for its analog functional modes and behavior throughout closing. For this, we initially focus on the actuation speed of the cFET. The proposed dynamic model follows the convention of first-order electrical systems analogies. This approach is well established in both general microfluidics^32^ and capillaric circuits^33^. Figure 2 shows the proposed model, overlaid on a rendering of the off*-*valve/cFET^22^.

In this model, there are two capillary pressures, symbolically modeled in Fig. 2a as voltages. The first pressure is created by the meniscus in the trigger channel, $P_{\rm{tr}}$. This is a constant value and can be calculated using Eq. 1. The second pressure, $P_{\rm{m}}$, is created by the meniscus that spans the large opening at the edge of the void volume. This pressure is not constant but rather a function of the displaced trigger channel volume, $V$. The magnitude of this pressure will increase at higher trigger channel displacements. Due to the complex geometry of the main channel, the main channel pressure cannot easily be calculated. However, *P*_m_ is expected to be similar in form to that shown in Fig. 2b.

There are four flow resistances that restrict fluid flow in this model. These represent the liquid and gas contributions for both the trigger channel and body of the main channel. Due to its large width, height, and short length compared to the trigger channel, the main channel flow resistances are assumed to be negligible.

It is worth noting that these flow resistances do not remain constant over the closing of the device. As the trigger channel fills with liquid, air is displaced, causing the liquid flow resistance to increase and the gas component to decrease. While for most cases, the air flow resistance is negligible compared to the liquid component, it cannot be neglected. If it was removed, then the total flow resistance would become 0 at $t = 0$. Using the two capillary pressures and the two trigger channel flow resistances, we can describe the flow rate in the trigger channel using a differential equation as

2 $$\dot V = \frac{{P_{\rm{tr}} - P_{\rm{m}}(V)}}{{R_{\rm{f,tr}}(V) + R_{\rm{g,tr}}(V)}},$$

where $\dot V$ is the first derivative of volume with time, $P_{\rm{tr}}$ is the pressure of the trigger channel, $P_{\rm{m}}(V)$ is the pressure of the main channel as a function of displaced trigger channel volume, $R_{\rm{f,tr}}(V)$ is the fluid resistance of the trigger channel as a function of displaced trigger channel volume, and $R_{\rm{g,tr}}(V)$ is the fluid resistance of the gas in the trigger channel as a function of volume. Equation 2 is linearly separable, so it should be possible to solve the differential equation analytically. However, as the analytical form of the main channel capillary pressure is not currently known, we proceed numerically in the following.

In our previous work^22^, the closing time of the off-valve (cFET) was measured by the time taken for the occluding bubble to touch the wall of the main channel. The volume of air required to do this was termed the “pinch-off volume”, $V_{\rm{p}}$. Therefore, the closing time of the valve is the time to displace $V_{\rm{p}}$ through the trigger channel. With initial conditions $V(0) = 0$ and $\dot V(0) = 0,$ we can express the closing time for a cFET as

3 $$t_{\rm{close}} = {\int}_0^{V_{\rm{p}}} {\frac{{R_{\rm{f,tr}}\left( V \right) + R_{\rm{g,tr}}(V)}}{{P_{\rm{tr}} - P_{\rm{m}}\left( V \right)}}dV}$$

Many of the parameters in Eq. 3 are known or can be calculated. The hydraulic resistance of a square cross-section channel, $R_{\rm{h}}$, can be calculated by the well-known empirical relation^34^,

4 $$R_{\rm{h}} \approx \frac{{12\,\mu L}}{{wh^3\left( {1 - 0.63\frac{h}{w}} \right)}},$$

where $\mu$ is the liquid viscosity, $L$ is the length of the channel, $w$ is the channel width, and $h$ is the channel depth. This can be used to calculate the trigger channel fluid resistances, $R_{\rm{f,tr}}$, and $R_{\rm{g,tr}}$. $P_{\rm{tr}}$ is defined by the trigger channel height, depth, and contact angle through the Young–Laplace equation. However, the pinch-off volume, $V_{\rm{p}}$, and the main channel capillary pressure are not known and are difficult to determine analytically.

**Fig. 3:**
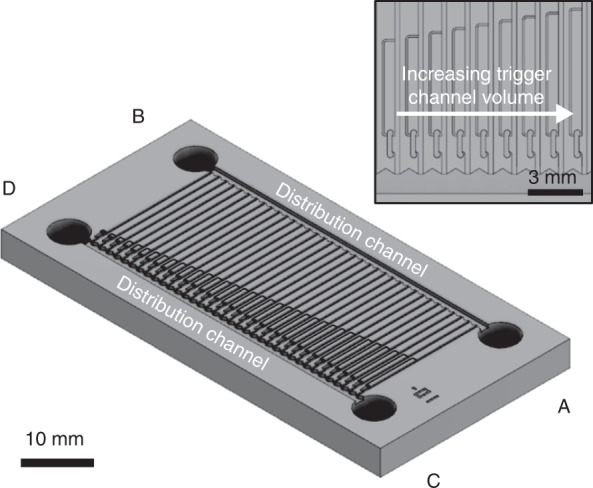
CAD rendering of the analog resistance mode test chip. The device was fabricated using micromilling in Polymethylmethacrylate, as reported previously^22^, and consisted of 36 cFET structures arranged in parallel between two large distribution channels with inlets A to D. The trigger channel volume of each cFET incrementally increases from right to left to create a full range of occluding bubble states. Inset shows a close-up illustrating how the trigger channel volume was incrementally increased by lengthening the trigger channels

**Fig. 4:**
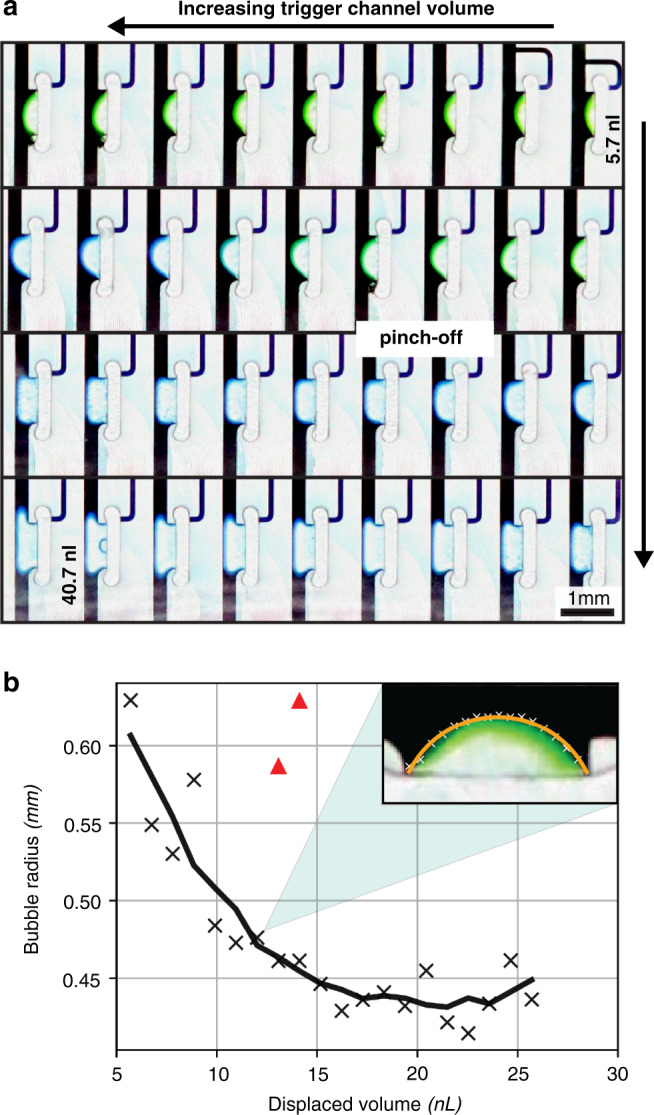
The radius of the occluding bubble, as determined by the test device. **a** Optical micrographs showing the shape of the occluding bubble for 36 cFETs with trigger channel volumes varying from 5.7 to 40.7 nL (top to bottom, right to left). Occluding bubbles contact the main channel wall, and thus pinch-off fluid flow, at a volume of approximately 26 nL. The shape of these bubbles was used to calculate main channel capillary pressure as function of displaced volume. **b** Plot of the occluding bubble radius as a function of displaced volume. A circular arc was fitted to each meniscus/bubble shown in **a** using a gradient descent fitting algorithm. Each point represents the radius of an arc that was fitted to the meniscus shape. Outliers are indicated as red triangles. Inset shows an example of the circular arc fitting (line) against the extracted meniscus shape (X), overlaid onto an image of the meniscus

To obtain values for these, a polymethylmethacrylate test chip was designed, fabricated, and tested (ESI, Fig. S1) according to methods described in detail in our previous work^22,26^. As per its design, this chip systematically varied the trigger channel volume of a number of cFETs to place the occluding bubbling in a range of states. These states are essentially stable snapshots of the meniscus at discrete time points throughout the complete closing of the cFET. A complete CAD rendering of the design is shown in Fig. 3 (CAD files provided in the ESI), with the inset depicting a close-up of the trigger channel structures. The variation in the channel length, and therefore volume, places the cFETs in a controlled range of states between completely open and completely closed. A full range of resultant bubble states can be seen in Fig. 4a. These states can in turn be used to determine both the pinch-off volume and the main channel capillary pressure. The pinch-off volume is estimated by visually inspecting when the bubble touches the opposite channel wall. In the case of the test chip, the trigger channel volume for which the bubble touches the main channel wall was determined to be 25.7 nL. This method of determining the pinch-off volume is limited by how accurately the point when the bubble touches the wall can be observed. In our previous work^22^, the ability to precisely determine this point was a limitation due to the resolution and frame rate of the recorded video. In this experiment, the resolution is still a limitation; however, because the displaced volume is static, the frame–rate error can be disregarded.

The main channel capillary pressure, on the other hand, has vertical and horizontal components. The vertical component is defined by the height and contact angle of the main channel and is constant throughout cFET closure. Meanwhile, the horizontal component can be calculated by finding the radius (principal radii) of the bubble in each subimage of Fig. 4a up until the pinch-off volume. Figure 4b shows the radii of the occluding bubbles plotted against the trigger channel volumes that created them until the pinch-off volume. An example of a circular arc fitted to the extracted meniscus shape is shown as an inset in Fig. 4b. In most cases, an excellent fit was achieved with a mean square error of less than 1 pixel. There were, however, two large outliers in the recorded data indicated in Fig. 4b as red triangles. In these cases, the meniscus did not pin the edges of the void volume due to a small leak. Despite this, there is a clear trend in the principal radii, and these outliers can be replaced with a linear interpolation of neighboring points.

Based on these data, Eq. 4 can now be evaluated for a range of contact angles between 0° and 60° and trigger channel heights from 20 to 150 μm. For calculation, the pinch-off volume was set to 25.7 nL, the trigger channel width to 100 μm, the trigger channel length to 10 mm, water as the working fluid (*η* = 8.9 × 10^−4^ Pa s, *γ* = 0.072 N/m), and air as the working gas (*η* = 1.872 × 10^−5^ Pa s)^35^. Main channel was 200 μm deep and 0.8 mm long at the large opening.

**Fig. 5:**
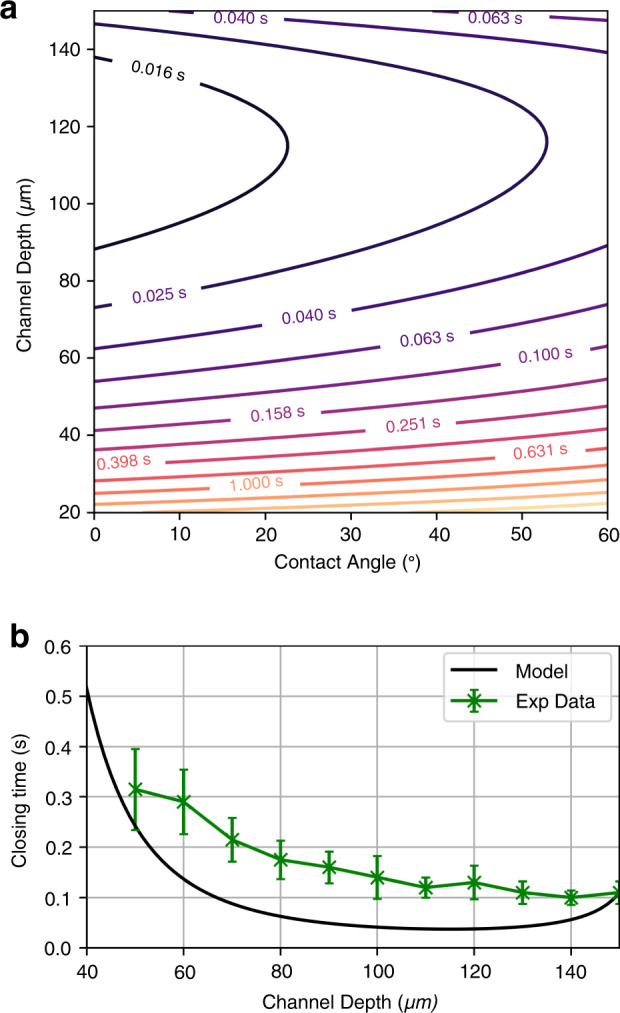
The closing time of the cFET as a function of trigger channel depth and water contact angle. **a** Contour plot of the closing time. The trigger channel depth was evaluated from 20 to 150 μm and the contact angle from 0° and 60°. For the main channel geometry in this example (200 μm deep) the minimum closing time is approximately 11 milliseconds. **b** Results of the modeled closing time, compared to the previously reported experimental results^22^

A contour plot of the closing time is shown in Fig. 5a. The minimum closing time for this cFET main channel geometry is 11 ms. This closing time is achieved at a contact angle of 0° with a trigger channel depth of 115.8 *μm* (width of 100 μm). As shown in Fig. 5, there is a significant basin of values where the closing time is on the order of 100 ms or less. This demonstrates the robustness of the general operation of the cFET, with even nonoptimal structures achieving good performance.

The results of the modeled closing time compared to the previously reported experimental results^22^ are shown in Fig. 5b. For a direct comparison, the model is recalculated for a trigger channel length of 36 mm and a contact angle of 0° to match the properties of the previous test device. While the agreement is reasonable, the model currently predicts generally faster closing than observed experimentally. In addition, the slowing of closing time due to high resistance or low capillary pressure trigger channels occurs at a higher rate than experimentally observed. This disagreement can be explained by fabrication variation in the chip, distension of the hydrophobic cover into the channel, or deviations in the liquid surface tension and viscosity due to the addition of dye and stabilizing agents; the first two can be reduced in the future through the use of more repeatable fabrication techniques such as injection molding and lamination.

It should also be stated that, due to the discrete size of the off*-*valves included on the test chip, the data presented in Fig. 5 currently do not fully examine the available parameter space. Further improvements to the slew rate or closing time of the valve could be made by adjusting other aspects of the valve geometry. For example, examining the effect of the main channel geometry on closing time could yield further advances. This strategy could be particularly impactful, as the length and depth of the main channel will change both the main channel capillary pressure and pinch-off volume. Unfortunately, the ability to optimize the main channel is currently limited due to the lack of a method to analytically determine the pinch-off volume and capillary back pressure. At present, any main channel geometry must first be physically fabricated, tested, and analyzed. However, as we demonstrate in the next section, this experimental approach still allows for a Shockley equation for cFETs to be developed.

### Analog transistor operation

#### Linear region

The linear or ohmic region of transistor behavior is the range of operation where the body of the transistor behaves similarly to a variable resistor. In the linear region, an increase in voltage across the drain to the source creates a linear and constant increase in the current flowing through the main body of the device due to ohmic conduction. Relating to the physical distribution of charge carriers in the device, the linear region starts when the channel is not modified by the depletion region at all, up to when the depletion region completely occludes the channel (pinch-off). The behavior of a transistor is described using the Shockley equation. This equation is one of several fundamental equations that characterize the behavior of electronic transistors^27,28^. It expresses the relationship between the current flowing through the main channel and the gate voltage applied. Due to the geometric parallelism that exists between electronic JFETs and off*-*valve-based cFETs, the Shockley equation should also describe the fluidic behavior of the cFET, albeit with minor adjustments.

In an electronic JFET, the depletion region volume is controlled by a potential energy (voltage) applied to the gate^27^. This is mirrored in the cFET by the position of the meniscus, which is controlled by the displaced volume in the trigger channel. Evidently, a cFET allows for volumetric fluid flow, rather than electronic current, to pass. For the cFET, a simplified Shockley equation can be modified to

5 $$Q_{\rm{D}} = Q_{\rm{ss}}\left( {1 - \frac{{V_{\rm{tr}}}}{{V_{\rm{p}}}}} \right)^2,$$

where *Q*_D_ is the flow rate through the main channel, *Q*_ss_ is the saturation flow rate, *V*_tr_ is the displaced trigger channel volume, and *V*_*p*_ is the pinch-off volume. As implied by *Q*_ss_ the original form of this Shockley equation is for the saturation region of transistor operation. Although initially we are examining the linear operation region, this form is useful because it excludes drain–source pressure and isolates the impact of the trigger channel volume. To determine the validity of this modified Shockley equation approach, the flow rate–volume (*Q*–V) relationship of the cFET was experimentally examined, demonstrating that analog resistance modes are controllable and achievable in this type of device.

To test the analog resistance modes, the same microfluidic device was used as for the characterization of the transient dynamics. This is possible, as each unique bubble volume creates a different fluidic resistance for liquid flowing through the main channel. When a hydrostatic pressure is applied across the cFETs, flow will be induced proportionally to the hydrostatic pressure applied and the size of the occluding bubble. On the test chip, the cFETs are arranged in parallel between two large distribution channels. The size of these distribution channels was chosen to minimize any driving pressure variation between transistors.

To initially fill the device, 105 µL of DI water, dyed blue, was loaded into one of the filling inlets (labeled C or D in Fig. 3) on the chip using a pipette. This filled the transistors with liquid and actuated the trigger channels for each device. The liquid did not flow into the top distribution channel because of the presence of a stop valve structure^1^, which terminates each parallel transistor branch. Following filling, all remaining liquid was pipetted out of the filling inlet reservoir. To initiate fluid flow, 105 µL of DI water dyed yellow was added to one of the testing inlets (labeled A or B in Fig. 3). Liquid then flowed through each transistor branch proportional to the resistance of each parallel branch.

To better understand the potential effect of the distribution channels, the filling–testing cycle was repeated multiple times along both ordinal axes of the chip. For this, the device was filled with blue dye-colored water either from inlet C and tested by adding yellow colored water to inlet B (*F*C*T*B) or from inlet D and tested by adding yellow colored water to inlet A (*F*D*T*A). The flow rate induced by the pressure was measured by recording the change in color through each transistor branch over time and subsequent analysis using ImageJ software. The flow rate measurement was made at the beginning of the experiment, before any significant backpressure could develop at the outlet. This visual method of tracking the liquid flow rate was used as a stand-in for a true volumetric flow rate measurement, which would be difficult to realize in the capillaric circuit.

**Fig. 6:**
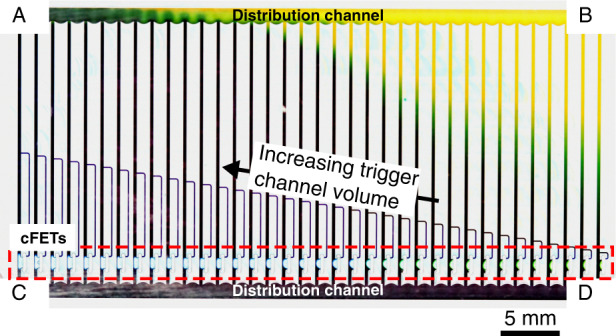
Photograph showing the total liquid flow through the parallel resistor branches in proportion to the fluid resistance of the cFET devices. Labels indicate the testing (A, B) and filling (C, D) inlets. In this example the device was filled with blue dye-colored water from inlet C and tested by adding yellow colored water to inlet B (FCTB). The image demonstrates the controllability in the resistance of the cFET devices

**Fig. 7:**
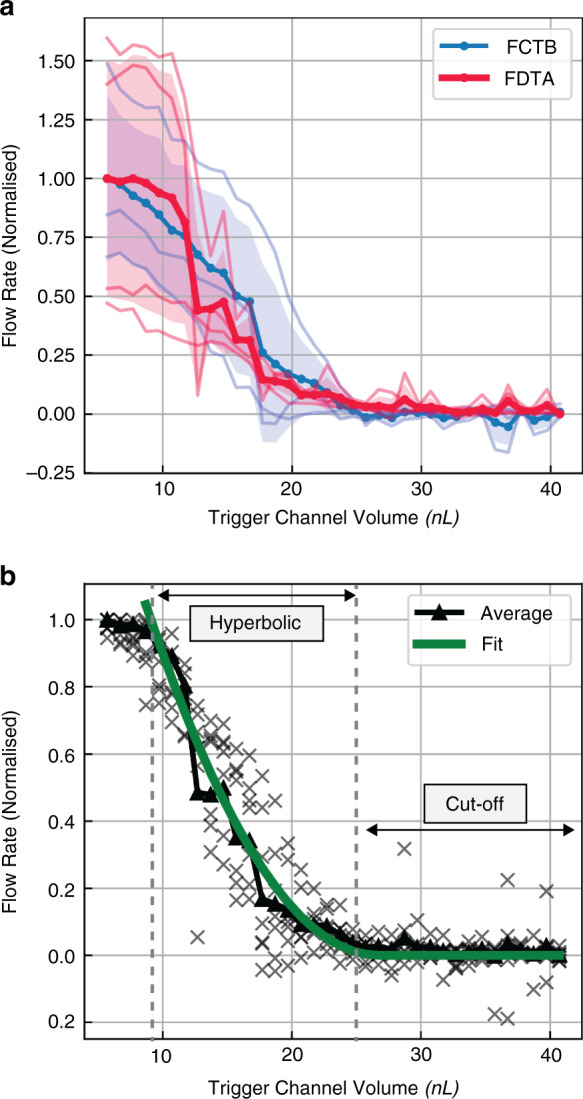
Flow rate–volume (*Q*–*V*) relationship for the cFET device. **a** Plot of the *Q*–*V* relationship for the cFET device, as measured from the test chip. The chip was tested with a hydrostatic pressure applied between diagonally opposite pairs of inlets. This was done to visualize any potential influence of the distribution channels. The results are shown normalized to the mean response to illustrate the effect of the trigger channel volume more clearly. Devices were filled from inlet C and tested via inlet B (*F*C*T*B), or filled from D and tested via A (*F*D*T*A). **b** A Shockley equation form fitted to the *Q*–*V* response shown in **a**

An example of such an experimental result can be seen in Fig. 6. Video footage of this *F*C*T*B and an example *F*D*T*A experiment are provided in the ESI (V1). Because the parallel branches filled at a different rate over the course of the experiment, they also filled to a different extent. As such, the progression of the yellow color along the individual parallel channels in Fig. 6 succinctly shows the controllability and general form of the cFET flow rate–trigger channel volume relationship. From these time-based data, *Q*–V plots (analogous to IV curves for a JFET) can be extracted, which are shown in Fig. 7a. In this graph, the results were normalized to the mean of each direction (FCTB, FDTA). This is because the hydrostatic driving pressure was uncontrolled in the experiment. This approach allows the effect of the trigger channel volume and flow resistances to be shown rather than the coupled effect of the trigger channels and driving pressure. In Fig. 7a, the *F*C*T*B test is shown in blue, and the *F*D*T*A test is shown in red. The mean of the recorded runs is illustrated as solid lines, with translucent lines indicating individual results, and the standard deviation of the measurement is given by the shaded regions.

The initial evaluation of Fig. 7a highlights two interesting phenomena. First, the hydraulic resistance of the off*-*valves is controlled by the applied trigger channel volume, in turn demonstrating that the analog resistance of the cFET can be controlled. Second, there are three distinct regions of operation reproduced on this chip design. For small trigger channel volumes, the fluid flow is mostly restricted by external resistances on the chip, for example, the resistances of the interconnecting channels. These are designed to be the same for all devices. For larger trigger channel volumes approaching the pinch-off volume, fluid flow is restricted by the increasing size of the occluding bubble. This creates a *hyperbolic* region where the flow resistance rapidly increases—a region described by the squared relationship of the Shockley equation (Eq. 5). On the other hand, for trigger channel volumes greater than the pinch-off volume, fluid flow in the cFETs is pinched off and restricted—this corresponds to a *cutoff* region. The *cutoff* region corresponds to a large, but finite, flow resistance level where fluid flow is almost completely restricted.

Figure 7b shows the modified Shockley equation fitted to the average of all the recorded data points. As illustrated by Fig. 7b, the Shockley equation provides a good fit for the experimental data in the *cutoff* and *hyperbolic* regions. In the low-volume regions, where the effect of external resistances is more pronounced, this fit breaks down, and the model and experimental data diverge. The point where the model diverges from the data is representative of when the external resistance becomes dominant over that of the cFET. This model fit also confirms the pinch-off volume estimation from Fig. 4a, with the flow rate dropping to zero after the observed pinch-off volume of 25.7 nL.

### Linear gain and transconductance parameter *g*_m_

Another key property of transistor action is the ability to amplify a signal. For a bipolar junction transistor^36^, it is possible to define a current amplification parameter, β, for an individual transistor. However, for a device that relies on a static applied field (continuous application of potential, rather than continuous application of current), such as an FET, the gain is expressed through the transconductance parameter *g*_m._ The transconductance (transfer conductance) expresses the change in conductance of a large-area channel in response to the applied field. Using this parameter, the voltage or current gain of a circuit can then be expressed using the relevant Shockley equation and passive circuit theory. In the case of the cFET, the data shown in Fig. 7 now allow for a transconductance to be defined and approximate values to be calculated. In an electronic JFET, the transconductance is the rate of change of the drain current, $I_{\rm{D}}$, with respect to the gate-source voltage, $V_{\rm{GS}}$, when the drain-source voltage is constant or the device is in saturation^37^. The equivalent fluidic transconductance parameter is the change in the main channel flow rate *Q*_m_ with respect to the change in the displaced trigger channel volume *V*_tr_ at a constant hydrostatic pressure. This corresponds to the partial derivative of Eq. 5 with respect to the displaced trigger channel volume. The maximum transconductance of this particular cFET main channel geometry was measured by fitting a linear line to the hyperbolic region shown in Fig. 7b), yielding $g_{\rm{m}} = 8.61 \pm 0.6\,{\rm{nLs}}^{ - 1}\,{\rm{nL}}^{ - 1}$ for the *F*D*T*A direction and $g_{\rm{m}} = 36.8 \pm 23.6\,{\rm{nLs}}^{ - 1}{\rm{nL}}^{ - 1}$ for the *F*C*T*B direction. The large variation between these results is because the device is currently operating in the linear regime, and the hydrostatic pressure is uncontrolled. The pressure driving flow motion in this experiment was a combination of hydrostatic pressure and the capillary pressure of the outlet meniscus, so it could not be assumed to be constant between experiments.

Interestingly, testing of the two diagonal directions on the chip (*FCTB, FDTA*) did not conclusively reveal an interfering effect due to the pressure drop along the distribution channel. Therefore, it was concluded that any potential effect of the distribution channel on the result was dominated by the variation in total driving pressure.

### Nonlinear flow response

Another important aspect of electronic transistor operation is the nonlinear flow regime called the saturation region. This region occurs when the voltage driving current through the conductive channel, *V*_*DS*,_ is comparable to the control voltage, *V*_GS_. In this circumstance, the driving voltage changes the shape of the depletion region, reducing its width. When *V*_DS_ *=V*_GS_, the conductive channel is completely pinched off; however, the current continues to flow due to ballistic transport. This effect means that the current in the device eventually saturates at a maximum value defined by the control voltage alone. This nonlinear conduction regime is extremely useful in electronics, as it facilitates the design of constant current supplies, as well as linear amplifiers. Such applications would also be of use in fluidic circuits for the creation of constant flow-rate pumps, which have been studied in capillary systems before^38,39^. These constant flow rate pumps allow reactants to be delivered at a constant rate, rather than the characteristic square root filling known in capillaric circuits^33^. A cFET-based realization of a constant flow rate pump would have benefits due to the smaller physical size and ability to programmatically adjust flow throughout the course of an experiment.

Although the ballistic transport responsible for conduction beyond pinch-off in JFETs does not exist for cFETs, the occluding bubble in the latter does experience distortion due to current flow in a similar manner to the depletion region in a JFET. As such, inspired by the potential applications, we characterized the flow mechanics of the cFET at larger driving pressures to determine the effect of bubble distortion on the main channel fluid flow.

**Fig. 8:**
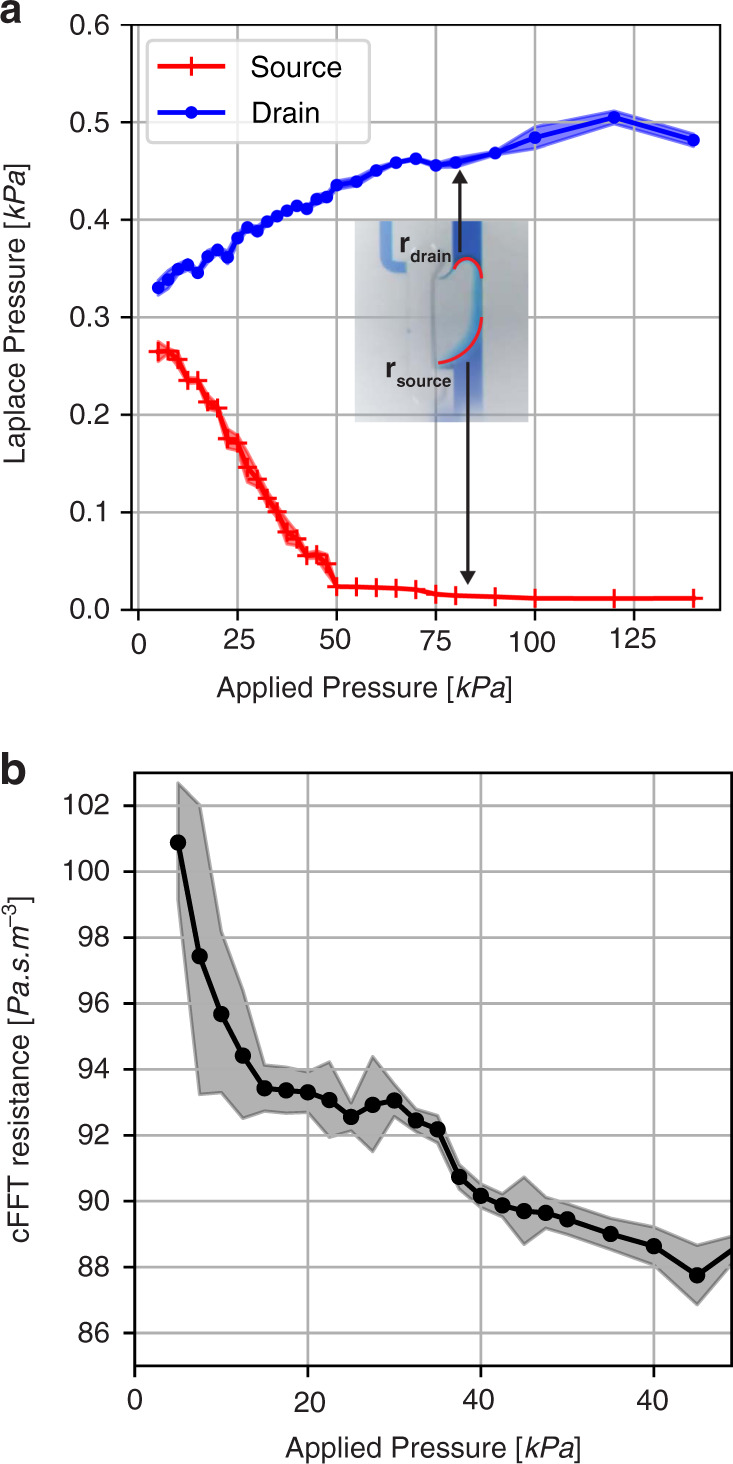
Nonlinear flow mechanics of a cFET under substantial applied pressure. **a** Laplace pressure, as derived from the bubble shape, of the source and the drain menisci as a function of applied pressure. Bubble movement modifies the restricted flow path, and therefore creates an overall nonlinear flow response. Inset: Representative image of the cFET when a strong pressure is applied. **b** Experimentally measured flow resistance of the cFET device under increasing pressure. The curve indicates nonlinear liquid conduction in the cFET due to the movement of the occluding bubble

Thus far, in this work, the applied driving pressure was small compared to the Laplace pressure of the occluding bubbles and was generally uncontrolled. As a result of the small magnitude, the pressure applied did not significantly distort the occluding bubble. Therefore, to examine the bubble distortion effect, a larger and more controllable pressure source was required. For this purpose, a conventional microfluidic pressure controller was utilized (Elveflow OB1 Mk3+) configured with a flow sensor (MFS-2, Elveflow). The test chip consisted of a single cFET device with its trigger channel directly connected to its drain such that it self-triggers upon filling. The volume of the occluding bubble was set to be above the pinch-off volume for this specific cFET main channel geometry. This allowed a well-controlled driving pressure to be applied to the occluding bubble while simultaneously measuring the resulting flow rate. The distortion of the occluding bubble was quantified by filming the cFET throughout the application of different pressures. Following this, the same nonlinear fitting process as in Fig. 4b was used to extract the radius of the menisci at the drain and source end of the cFET and to calculate the horizontal component of Laplace pressure for each. The Laplace pressure of the bubble, derived as such from its shape, is shown in Fig. 8a, while the corresponding flow resistance of the cFET as a function of driving pressure is shown in Fig. 8b.

Figure 8a demonstrates that as pressure is applied, the radius of the meniscus at the source end of the cFET increases, while the radius of the drain end tightens. This bubble movement means that the source meniscus is partially displaced into the body of the cFET (Fig. 8a inset). This displacement increases the average cross-section of the flow path in the main channel and as such reduces the flow resistance of the device. The flow resistance of the cFET can be calculated using the applied pressure and measured resulting flow rate, and this result is shown in Fig. 8b. This demonstrates that as the driving pressure increases, the resistance of the cFET decreases in a nonlinear manner. As such, it can be concluded that this cFET design did indeed exhibit a nonlinear flow response because of self-feedback. However, it should be noted that this response is characteristic of a yielding mechanism rather than a saturating behavior. In the absence of external resistances, a yielding nonlinear flow response will create an unstable system, making this a not immediately useful property. Nonetheless, because the nonlinear flow resistance of the cFET is caused by changes in occluding bubble shape, the existence of a self-feedback mechanism should allow the response type to be tuned by adjusting the shape of the main channel. For example, a constriction could be added to the drain of the cFET, which would increase fluid resistance when the bubble is displaced into it. This may potentially create a saturation response, albeit one that is geometric in origin.

In addition to this exploration of the nonlinear flow mechanisms in the cFET, a test chip to characterize the in-circuit flow behavior for pinched-off cFETs was fabricated and tested (see ESI, Fig. S4). Interestingly, the in-circuit behavior created a constant drain velocity, which was initially hypothesized. While this initially seems contrary to the results shown in Fig. 8b), it is believed to be caused by the cFET leakage resistance dominating the total circuit resistance and therefore creating an apparent constant flow rate. While this is a practical and useful result, it is not a manifestation of nonlinear flow mechanics, as it is an extension of the analog resistance mode region already discussed via the finite off-resistance in the pinched off regions.

### Reversible operation of the cFET

The second and final criterion on which the original off*-*valves fell short of true transistor analogy was their ability to oscillate. Capillary action devices are generally single use: Once a channel has been filled, the potential energy of the system has been expended, and no more liquid actuation can occur^31^. This in turn means that reversibility of any action within the system is difficult, if not impossible, and that reopening of the cFET by simply withdrawing liquid from the trigger channel will be difficult.

Interestingly, we found that the cFET can in fact operate reversibly, so long as there is still potential energy in the capillaric circuit and there is feedback void volume. This is possible because the bubble in the main channel of the device, in the closed or partially closed state, has its own capillary pressure. As such, the capillary pressure of the occluding bubble constitutes a restoring force that is balanced by the trigger channel pressure. If the capillary pressure of the trigger channel is mitigated, the volume is retracted, or if a vent is made available to the void volume, the cFET will reopen.

In this work, we present the simplest possible demonstration of this operation in the form of a vent being opened to the void volume of the transistor, allowing the cFET to reopen under its own restoring force. The chip utilized to experimentally demonstrate this was a modified version of a multiple-input NAND device reported previously (CAD files provided in the ESI)^26^. This device consists of multiple trigger channels that lead to a single void volume. In its ordinary operation, the valve operates like a computational NAND function, completely closing only when all the trigger channels have been actuated^26^.

**Fig. 9:**
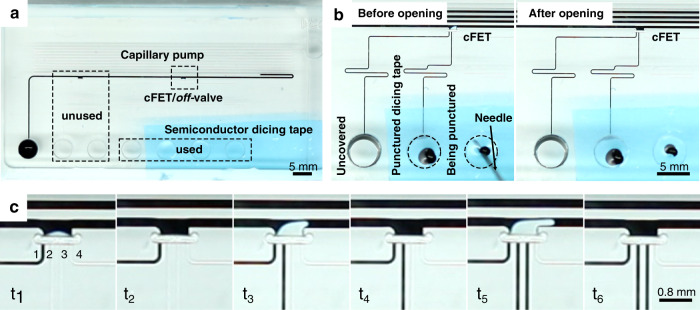
Demonstration of the closing and reopening of a cFET device by venting of the void volume. **a** Photograph of the capillary logic chip design^26^ used for the reopening demonstration. A capillary pump actuates fluid flow through two off-valves/cFETs of which only the second valve is used for the demonstration. This second cFET has four trigger channels with externally accessible inlets. Three of these inlets are closed off with semiconductor dicing tape, providing an airtight seal. **b** Example of the switching between closed and reopened cFET states. Filled trigger channels 1 and 2 expand a bubble into the main channel (left). After the dicing tape covering channel 3 is punctured the bubble recovers, reopening the cFET (right). **c** Image sequence showing the bubble expansion and retraction over three transitions. At $t_1$ the cFET is shown in the closed state with only one trigger channel providing the volume. When trigger channel 2 is vented at $t_2 > t_1$ the cFET reopens. At $t_3$ the cFET is closed again with the help of trigger channel 2, and reopened at $t_4$by piercing the tape membrane over trigger channel 3. This transition is repeated a third time at $t_5$, closing the cFET again with the help of trigger channel 3, and reopened it at $t_6$ via trigger channel 4. While the main purpose of this experiment was to show that the occluding bubble has a restoring force, and is able to reopen if required, it also illustrates that the volume of the actuating bubble is an adjustable function of the number of trigger channels

For the demonstration of reopening, the inlets to all but one of the trigger channels were masked with semiconductor dicing tape, with the tape acting as a burstable membrane. In this configuration, any of the trigger channels will actuate the main channel of the cFET. Once the valve has been closed, a user then bursts one of the tape seals, for example, by manually puncturing it with a syringe needle. The void volume is now able to vent through the burst membrane, and the restoring force created by the main channel meniscus will reopen the device. An experimental demonstration of this operation can be seen in Fig. 9. Video footage of this experiment is provided in the ESI (V2). At time point *t*_1_, the cFET was closed, with one trigger channel filled. This was followed by puncturing of the dicing tape on the inlet of channel 2 at *t*_2_, leading to valve reopening. At *t*_3_, trigger channel 2 was filled to reclose the valve and reopened by puncturing the tape over channel 3 in *t*_4_. This manual cycling was repeated one more time during *t*_5_ and *t*_6_, with the possibility of extending this for as many times as required by the application. The total number of possible switching events is limited only by the number of vent channels that can physically be routed to the void volume.

Although this demonstration is relatively crude, it functionally demonstrates the reversibility of the cFET operation, providing a novel flow-control pathway for relevant applications. For example, many biochemical assays require timed incubation steps^22^. Capillaric circuits generally manage the timing of incubation steps by use of long delay channels^2,40,41^; however, it may also be desirable to have user-controlled incubation periods. In such an application, the cFET would be used to stop liquid progress using a feedback trigger channel, and a user would manually resume fluid flow by bursting the tape membrane to vent the void volume. Evidently, there are other capillaric circuit elements that can also achieve such user actuated timing, the simplest example being a two-level trigger valve^1^. However, the potential advantage of using a cFET over a two-level trigger valve in this application would be for stability over longer incubation periods. For example, we have observed that the closed state of cFETs tends to be stable for 2 hours or more (see ESI, Fig. S2), which is significantly longer than the 15–30 min reported for conventional trigger valves^42^.

Ultimately, though, as the goal of capillary circuits typically is to provide fully autonomous function, the integration of mechanically moving parts, such as burstable membranes, is not necessarily a good fit due to their need for user intervention. Despite this, the experiments shown here conclusively demonstrate that the off*-*valve/cFET structure is fully capable of oscillation given an appropriate input. It would be conceivable to create automatic methods to achieve the same result using supporting capillaric circuitry. These could include using a capillary action retention valve^1^ to remove liquid out of the trigger channel and connecting a lower flow resistance pump to the trigger channel.

## Discussion

In this paper, we have reported the first study of analog resistance modes of a capillaric off*-*valve, providing support for the introduction of the term capillaric FET, or cFET in short, for this type of structure. The capability of a cFET to actively control the flow resistance of a channel, as shown, represents a substantial functional addition to the capillaric circuit tool kit. Indeed, a wide range of applications in capillarics could profit from this kind of flow control. For example, the cFET could be used to control the stoichiometry of flowing reactants in a microfluidic channel, as already demonstrated in pressure-driven systems^24^. Traditionally, this would be controlled by the concentration of the input reactants or by designing the size of the microchannels to obtain a specific flow resistance^43^. A cFET could not only achieve the same result as most of these solutions, but, more importantly, it could do so while also changing its resistance over time as part of a program hard-coded into the circuit. For example, if the cFET was open initially and then actuated later in time, the ratio of reactants could be changed in a ramp or step function-like manner.

Based on the data shown here, natural limitations to the interoperability of the analog resistance modes demonstrated remain. Currently, the *Q*–*V* curve experiment shown in Fig. 7a does not control the hydrostatic pressure applied across the device as precisely as would be desirable. These results allowed us to conclude that the occluding bubble can be placed in a variety of states, thus producing a useful difference in flow resistance due to the pressure drop along the distribution channel and the driving pressure variation. We were not able to recreate a *Q*–*V* plot for a single device in isolation. Future work will thus seek to extend the physical characterization of the structure and modify the main channel geometry. In particular, we aim to precisely measure the nonlinear flow characteristics of the device over a wider range of trigger channel volumes and use these results to modify the channel geometry to create flow saturating behavior^24^. Further study and prediction of the analog resistance modes of the cFET will establish this new type of flow-control device for a whole range of practical applications, thus enabling the realization of previously incompatible assays on capillaric microfluidics platforms.

## Materials and methods

### Fabrication

Devices were fabricated from cross-linked polymethylmethacrylate (PMMA; 4.5 mm general purpose acrylic; PSP Plastics, Christchurch, New Zealand) as previously described^22,26^. Channel milling was performed using a Mini-Mill/GX micromilling machine running an NSK-3000 Spindle (Minitech Machinery Corporation, Norcross, GA, USA) with a minimum addressable step size of 1 µm. Machining tools were purchased from Performance Micro Tool (Janesville, WI, USA) in diameters of 3.175 mm (SR-4-1250-S), 250 µm (250M2X750S) and 100 µm (100M2X300S) for the square heads and 200 µm (TR-2-0080-BN) for the ball nose. Design files and milling parameters (G-code) were prepared using computer-aided design (CAD) software (Autodesk Fusion 360^©^ 2020 Autodesk, Version 2.0.10356) for all functional units (CAD files provided in the ESI).

Each sample was fabricated by an initial face cut (3.175 mm cutter) to level out the surface, followed by milling of each channel. Milling of shallower channels was carried out first to avoid burring on the edges, and all channel steps were repeated at the bottom height at the end of milling to remove burrs. The surface was then polished using acrylic polish (aluminum oxide-based CRC, code 9230), followed by ultrasonication for 1 min in ~5% (v/v) aqueous isopropyl alcohol solution, washing with acetone, isopropyl alcohol and water and blow drying with nitrogen. To close microscopic cracks that arose during the milling process, the surface was coated with high molecular weight PMMA solution (average Mw = 996,000, 2.5% in xylene; Sigma Aldrich, St Louis, MO, USA). Any remaining solvent was removed by drying the samples at 90 °C for 5 min on a hotplate and keeping the hot sample under vacuum for at least 1 min. Finally, the samples were plasma-treated ten times for 1 min, each time at 25 W, in pulsed mode (ratio 50) using oxygen gas (3 sccm; Tergeo Plasma Cleaner, Pie Scientific, Union City, CA, USA). A thin (2–3 mm) polydimethylsiloxane slab was prepared (PDMS; Sylgard 184, Electropar, NZ; mixed as given in the instructions (10:1 w-w base:curing agent) and then cured at 80 °C for 2 h). This PDMS slab acted as a hydrophobic cover for the chip. A frame holder was used to ensure a tight seal of this to the PMMA channels. For the demonstration of reopening, inlets on the PMMA were masked with semiconductor dicing tape (SWR 10+R, Nitto) and pierced with syringe needles.

### Device testing

All flow experiments were conducted by the addition of aqueous dye solutions (Brilliant Blue dye, #80717, Sigma Aldrich and Tartrazine, #T0388, Sigma Aldrich) into the reservoirs using a manual pipette or, for the nonlinear flow response experiment, directly into the main channel using a pressure controller (OB1 Mk3+, Elveflow). In the latter case, flow was delivered using tubing (1516 L, Kinesys) and measured using an inline flow sensor (MFS-2, Elveflow). Liquid movement was recorded using a digital camera (Canon EOS 760D using a Canon Macro lens EF 100 mm 1:2.8 USM, recorded at 25 FPS). Images of the bubble shapes were binarized using ImageJ (FIJI)^44^ and used to extract the shape of the bubble. A multivariate gradient descent fitting algorithm could then be used to fit the origin and radius of a circular arc to the bubble. All data processing was performed using ImageJ and Python 3.8^45^ (Code provided in the ESI).

## Supplementary information

Electronic Supplementary Information

CAD File 1

CAD File 2

CAD File 3

CAD File 4

Video 1

Video 2

## References

[1] Safavieh R, Juncker D (2013). Capillarics: pre-programmed, self-powered microfluidic circuits built from capillary elements. Lab Chip.

[2] Olanrewaju A, Beaugrand M, Yafia M, Juncker D (2018). Capillary microfluidics in microchannels: from microfluidic networks to capillaric circuits. Lab Chip.

[3] Juncker D (2002). Autonomous microfluidic capillary system. Anal. Chem..

[4] Park J, Han DH, Park J-K (2020). Towards practical sample preparation in point-of-care testing: user-friendly microfluidic devices. Lab Chip.

[5] Sachdeva S, Davis RW, Saha AK (2021). Microfluidic point-of-care testing: commercial landscape and future directions. Front. Bioeng. Biotechnol.

[6] Salva ML, Rocca M, Hu Y, Delamarche E, Niemeyer CM (2020). Complex nucleic acid hybridization reactions inside capillary-driven microfluidic chips. Small.

[7] Vinitha TU, Ghosh S, Milleman A, Nguyen T, Ahn CH (2020). A new polymer lab-on-a-chip (LOC) based on a microfluidic capillary flow assay (MCFA) for detecting unbound cortisol in saliva. Lab Chip.

[8] Li Y (2021). A distance-based capillary biosensor using wettability alteration. Lab Chip.

[9] Ma B (2020). Wearable capillary microfluidics for continuous perspiration sensing. Talanta.

[10] Hemmig E, Temiz Y, Gökçe O, Lovchik RD, Delamarche E (2020). Transposing lateral flow immunoassays to capillary-driven microfluidics using self-coalescence modules and capillary-assembled receptor carriers. Anal. Chem..

[11] Hassan S-u, Zhang X (2020). Design and fabrication of capillary-driven flow device for point-of-care diagnostics. Biosensors.

[12] Hassan S-u (2020). Capillary-driven flow microfluidics combined with smartphone detection: an emerging tool for point-of-care diagnostics. Diagnostics.

[13] Ghosh S (2020). A new microchannel capillary flow assay (MCFA) platform with lyophilized chemiluminescence reagents for a smartphone-based POCT detecting malaria. Microsys. Nanoeng..

[14] Che C (2019). Activate capture and digital counting (AC + DC) assay for protein biomarker detection integrated with a self-powered microfluidic cartridge. Lab Chip.

[15] Epifania R, Soares RRG, Pinto IF, Chu V, Conde JP (2018). Capillary-driven microfluidic device with integrated nanoporous microbeads for ultrarapid biosensing assays. Sens. Actuators B.

[16] Olanrewaju AO, Ng A, DeCorwin-Martin P, Robillard A, Juncker D (2017). Microfluidic capillaric circuit for rapid and facile bacteria detection. Anal. Chem..

[17] Liu D (2017). A fully integrated distance readout ELISA-Chip for point-of-care testing with sample-in-answer-out capability. Biosens. Bioelectron..

[18] Delamarche E, Temiz Y, Lovchik RD, Christiansen MG, Schuerle S (2021). Capillary microfluidics for monitoring medication adherence. Ang. Chem. Int. Ed.

[19] Olanrewaju AO, Robillard A, Dagher M, Juncker D (2016). Autonomous microfluidic capillaric circuits replicated from 3D-printed molds. Lab Chip.

[20] Hitzbleck M (2012). Capillary soft valves for microfluidics. Lab Chip.

[21] Arango Y, Temiz Y, Gökçe O, Delamarche E (2020). Electro-actuated valves and self-vented channels enable programmable flow control and monitoring in capillary-driven microfluidics. Sci. Adv..

[22] Menges J (2020). New flow control systems in capillarics: off valves. Lab Chip.

[23] Gao Y, Wu M, Lin Y, Xu J (2020). Trapping and control of bubbles in various microfluidic applications. Lab Chip.

[24] Khoshmanesh K (2015). A multi-functional bubble-based microfluidic system. Sci. Rep..

[25] Zhou Y (2020). Standing air bubble-based micro-hydraulic capacitors for flow stabilization in syringe pump-driven systems. Micromachines.

[26] Meffan, C. et al. Transistor off-valve based feedback, metering and logic operations in capillary microfluidics. In: IEEE 34th International Conference on Micro Electro Mechanical Systems (MEMS), Virtual, 218–221 (IEEE, 2021).

[27] Shockley W (1952). A unipolar “Field-Effect” transistor. Proc. IRE.

[28] Dacey GC, Ross IM (1953). Unipolar “Field-Effect” transistor. Proc. IRE.

[29] Vourdas N, Moschou DC, Papadopoulos KA, Davazoglou D, Stathopoulos VN (2018). A new microfluidic pressure-controlled Field Effect Transistor (pFET) in digital fluidic switch operation mode. Microelectron. Eng..

[30] de Wijs W-JA, Laven J, de With G (2016). Wetting forces and meniscus pinning at geometrical edges. AIChE J..

[31] Narayanamurthy V (2020). Advances in passively driven microfluidics and lab-on-chip devices: a comprehensive literature review and patent analysis. RSC Adv..

[32] Kirby, B. *Micro- and Nanoscale Fluid Mechanics: Transport in Microfluidic Devices* (Cambridge University Press, 2010).

[33] Mikaelian D, Jones B (2020). Modeling of capillary-driven microfluidic networks using electric circuit analogy. SN Appl. Sci..

[34] Bruus, H. *Theoretical Microfluidics* (Oxford Univ. Press, 2008).

[35] Rumble, J. (ed) *CRC Handbook of Chemistry and Physics* (CRC Press Boca Raton, 2020).

[36] Shockley W (1949). The theory of p-n junctions in semiconductors and p-n junction transistors. Bell Syst. Tech. J..

[37] Sedra, A. S. & Smith, K. C. *Microelectronic Circuits* (Oxford Univ. Press, 2015).

[38] van der Wijngaart W (2014). Capillary pumps with constant flow rate. Microfluid. Nanofluid..

[39] Guo W, Hansson J, van der Wijngaart W (2018). Capillary pumping independent of the liquid surface energy and viscosity. Microsyst. Nanoeng..

[40] Gervais L, Delamarche E (2009). Toward one-step point-of-care immunodiagnostics using capillary-driven microfluidics and PDMS substrates. Lab Chip.

[41] Gervais L, Hitzbleck M, Delamarche E (2011). Capillary-driven multiparametric microfluidic chips for one-step immunoassays. Biosens. Bioelectron..

[42] Zimmermann M, Hunziker P, Delamarche E (2008). Valves for autonomous capillary systems. Microfluid. Nanofluid..

[43] deMello AJ (2006). Control and detection of chemical reactions in microfluidic systems. Nature.

[44] Schindelin J (2012). Fiji: an open-source platform for biological-image analysis. Nat. Meth.

[45] Rossum, G. V. & Drake, F. L. *Python 3 Reference Manual* (CreateSpace, 2009).

