# Supplementary material for: Capillaric field effect transistors
Source: Microsyst Nanoeng. 2022 Mar 21;8:33. doi: 10.1038/s41378-022-00360-8 (PMC8934874; doi:10.1038/s41378-022-00360-8)
Supplement: Supplementary file 1 — Electronic Supplementary Information [file 41378_2022_360_MOESM1_ESM.pdf]

Capillaric-Field Effect Transistors

Claude Meffan*[abf†] , Julian Menges[ab] , Fabian Dolamore[bc], Daniel Mak[ab], Conan Fee[c], Renwick C.J. Dobson[bde] and Volker Nock*[ae†]

[a]Department of Electrical and Computer Engineering, University of Canterbury, Christchurch, New Zealand.
[b]School of Biological Sciences, University of Canterbury, Christchurch, New Zealand.
[c]School of Product Design, University of Canterbury, Christchurch, New Zealand.
[d]Department of Biochemistry and Molecular Biology, Bio21 Molecular Science and Biotechnology Institute, University of Melbourne, Victoria, Australia.
[e]MacDiarmid Institute for Advanced Materials and Nanotechnology, Wellington, New Zealand
[f]Department of Micro engineering, Kyoto University, 615-8540, Kyoto, Japan
[†]Correspondence to Claude Meffan claude.meffan@canterbury.ac.nz or Volker Nock, volker.nock@canterbury.ac.nz

List of contents:

Part I: Supplementary figures S1, S2, S3, S4.

Part II: Supplementary code

Part III: Supplementary movies V1 & V2

Part IV: Supplementary CAD files CAD1 & CAD2

Part I: Supplementary figures

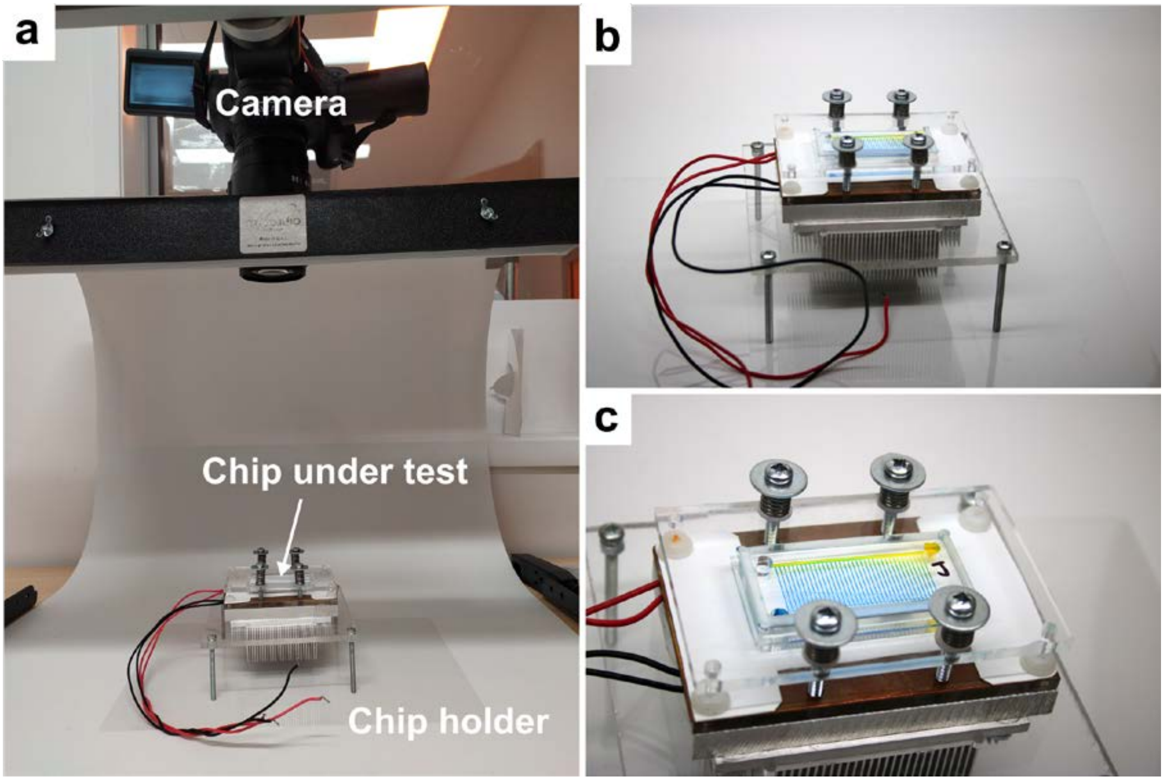

Figure S1: Experimental setup. a Photograph of the custom-made chip holder and chip under test in the photo booth (PS5, MyStudio) used to record cFET characteristics. The camera was a digital single-lens reflex camera (DSLR) Canon EOS760D with a Canon macro lens EF 100 mm 1:2.8 USM and videos were recorded at 25 FPS in .mp4 format. **b** Close-up photograph of a test chip in the chip holder. Note: Although the holder enables temperature control, this feature was not used during the experiments, indicating the robustness of the flow control. **c** Four screws with compression springs and a PMMA frame were used to provide repeatable clamping pressure to the stack of PMMA chip, flat piece of polydimethylsiloxane (PDMS) and PMMA baking blank.

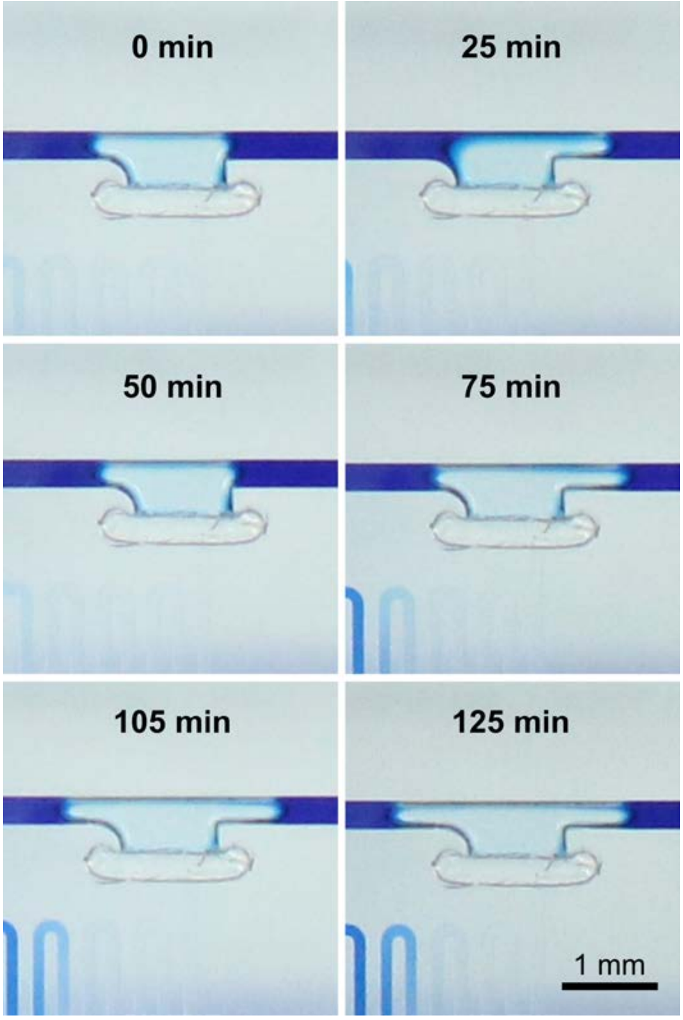

Figure S2: Example of *off*-valve/cFET state stability. Once inflated via the trigger channel, bubbles blocking main channel flow remain stable over a period in excess of 2 hours. While bubbles may move and change shape over time due to thermal fluctuations or evaporation from chip openings, they remain pinned on the void volume. Note: Neither temperature, nor evaporation out of the chip inlets or outlets were controlled for this experiment.

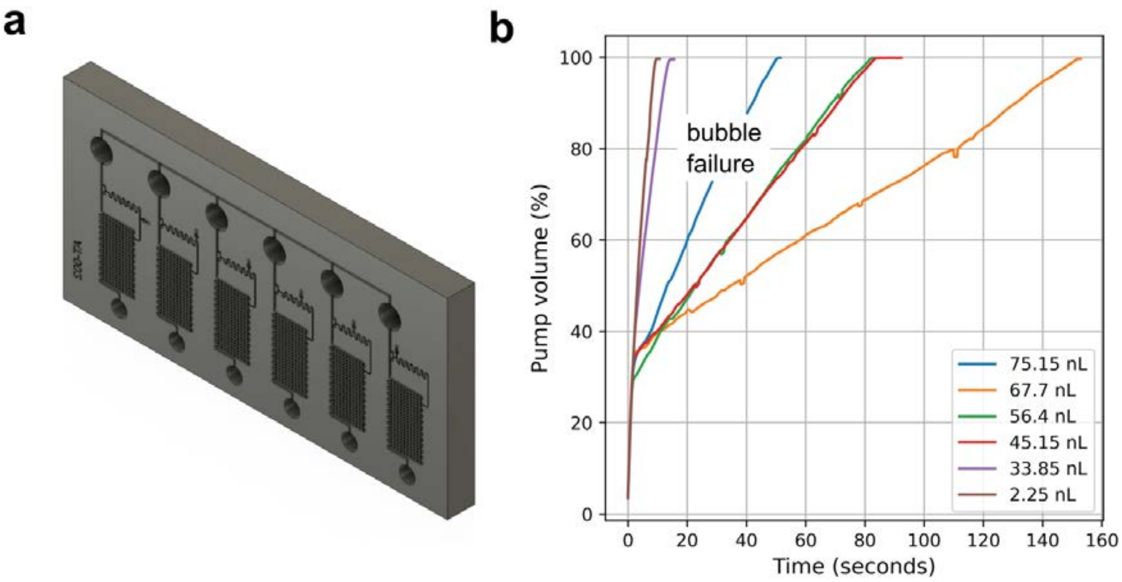

Figure S3: Fluid flow beyond pinch-off. a CAD rendering of the test chip. Six cFETs with varied trigger channel volumes were connected to capillary pumps with capillary pressures above that of the occluding bubbles meniscus. This meant fluid flow continued beyond pinch-off. **b** Displaced fluid in the pump as a function of time. Total displacement is given as a percentage of total pump volume. After each cFET was actuated, the flow in the pump did no longer follow the characteristic square-root filling behaviour normally observed in capillary systems. The observed constant flow rate behaviour is believed to be due to the resistance of the cFET becoming the dominating value in the system, as opposed to a velocity saturation effect. This cFET resistance was clearly still controllable via the bubble volume. At the largest volume investigated, the bubble failed and was displaced into the pump. This created the reduced resistance observed in the 75.15 nL result.

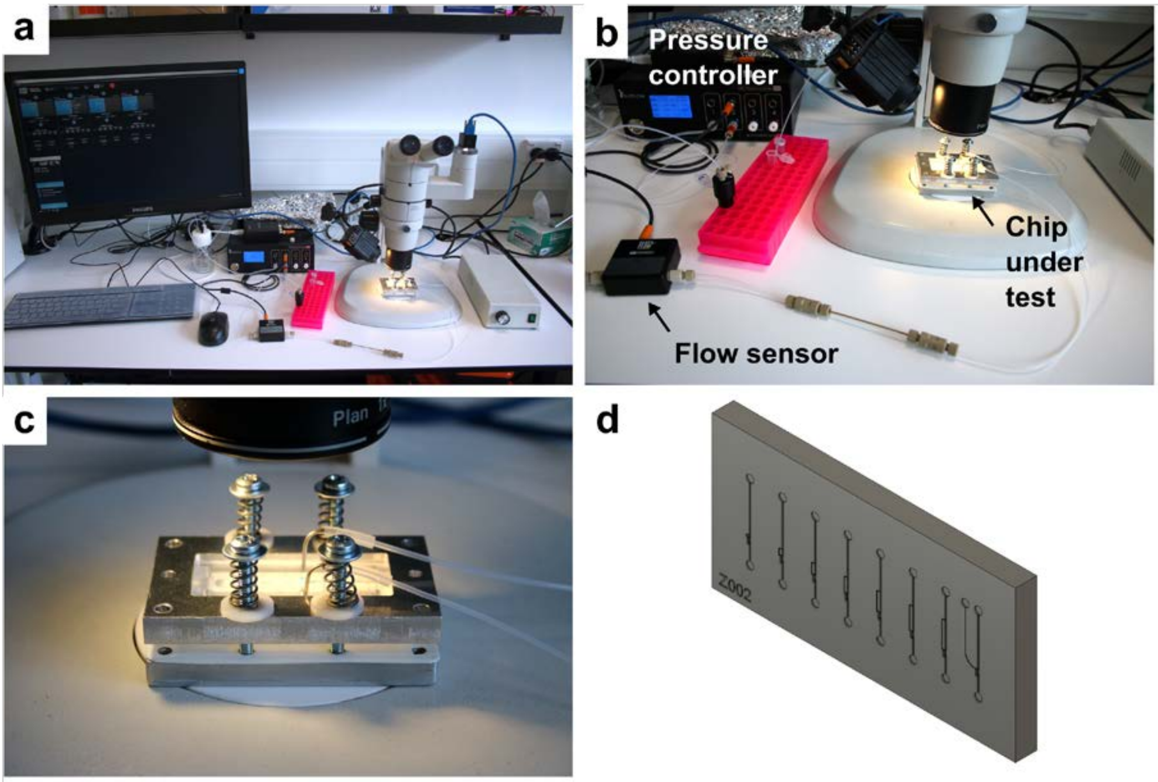

Figure S4: Pressure driven flow experiment apparatus. To analyse the cFETs response to larger driving pressures, an external pressure controller (Elveflow OB1 Mk3+), in combination with a flow sensor (MFS-2, Elveflow) was utilised. **a** Photograph showing the experimental setup including the control PC and inspection microscope and camera used to record the cFET behaviour. **b** Close-up of a test microfluidic device configured to interface with the pressure controller via a flow sensor and external tubing. **c** Close-up photograph of the cFET chip device in a custom holder clamping the PMMA chip and flat PDMS cover. The latter had 1 mm diameter Through-holes cored into it to connect tubing. **d** CAD model of the test chip, which contained individual cFET devices configured to self-trigger (trigger channel directly connected to drain terminal).

Part II: Supplementary code

cfet-closing-time-model: The model evaluates the closing time for a capillaric field effect transistor for a range of trigger channel depths and contact angles.

Available from https://github.com/claudemeffan/cfet-closing-time-model

Part III: Supplementary movies

Movie V1: "Analog resistance modes of the cFET" contains video results of two experiments used to determine the Shockley equation equivalent for capillaric-FETs. Total liquid flow through the parallel resistor branches is proportional to the fluid resistance of the cFET device, demonstrating the controllability of the resistance with the device. Two test configurations are shown, one clip of a fill C, test B (*F*C*T*B) and a second clip of a fill D, test A (*F*D*T*A). Videos were recorded using a Canon EOS760D and a Canon macro lens EF 100 mm 1:2.8 USM at 25 FPS.

Movie V2: "*Off*-valve/cFET reopening and closing" contains the video results showing the reversible operation of an *off*-valve/cFET. External inputs covered with semiconductor dicing tape (SWT 10+R, Nitto) are used to manually close and reopen a valve, providing a simple demonstration that the cFET structure is capable of oscillating. Videos were recorded using a Canon EOS760D and a Canon macro lens EF 100 mm 1:2.8 USM at 25 FPS.

Part IV: Supplementary CAD files

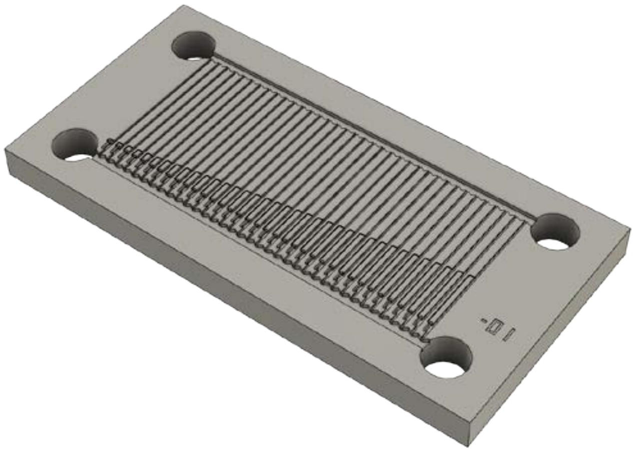

File CAD 1: “Transient dynamics of the cFET” contains the chip design used to characterise the transient dynamics and Shockley equation equivalent of the cFET described in Figs. 3-7 in .STEP format.

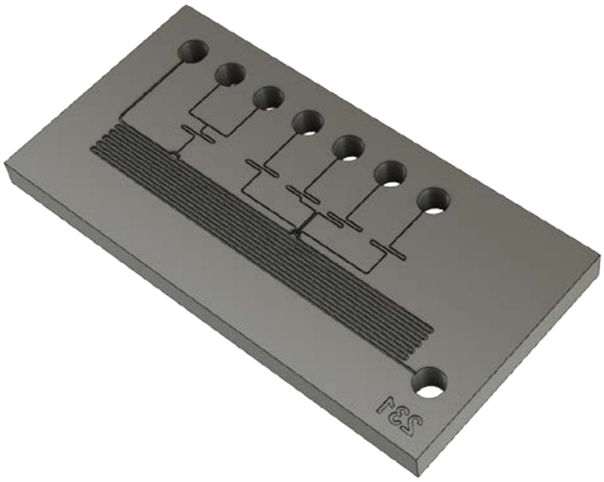

File CAD 2: “Reversible operation of the cFET” contains the chip design used to demonstrate reversible operation of the cFET described in Fig. 9 in .STEP format.

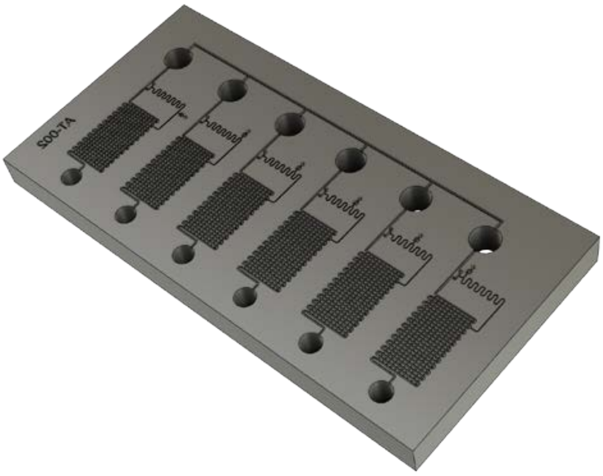

File CAD 3: “Fluid flow beyond pinch-off” contains the chip design used to analyse the cFETs response beyond pinch-off described in Fig. S3 in .STEP format.

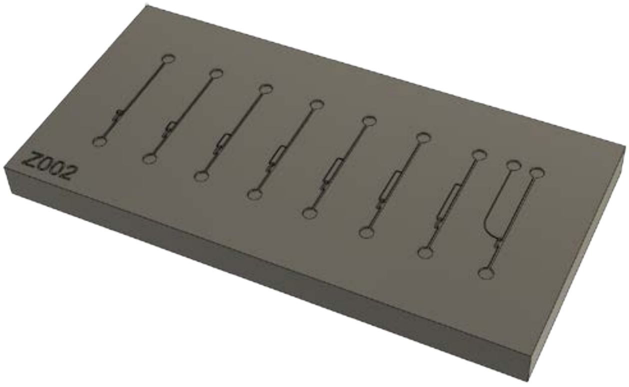

File CAD 4: “Pressure-driven cFET flow experiment” contains the chip design used to analyse the cFETs response to larger driving pressures described in Figs. 8 and S4 in .STEP format.
